# Thrombotic outcomes and mortality with roxadustat for anemia in chronic kidney disease: a systematic review and meta-analysis of randomized trials

**DOI:** 10.3389/fphar.2026.1792709

**Published:** 2026-06-10

**Authors:** Jingyi Tang, Yu Zheng, Lihua Pan, Xueling Li, Yifei Zhong

**Affiliations:** 1 Department of Nephrology, Longhua Hospital Shanghai University of Traditional Chinese Medicine, Shanghai, China; 2 Department of General Practice, Hangtou Hesha Community Health Service Center, Shanghai, China

**Keywords:** anemia, chronic kidney disease, meta-analysis, mortality, roxadustat, vascular access thrombosis, venous thromboembolism

## Abstract

**Background:**

Roxadustat is an oral hypoxia-inducible factor prolyl hydroxylase inhibitor used to treat anemia in patients with chronic kidney disease (CKD); however, evidence from randomized trials has not fully clarified its associations with thrombotic outcomes and all-cause mortality.

**Methods:**

We searched PubMed, Embase, Web of Science, and the Cochrane Library from inception to 21 August 2025 for randomized controlled trials comparing roxadustat with placebo or erythropoiesis-stimulating agents (ESAs) in adults with CKD. The primary outcome was vascular access thrombosis (VAT), while the secondary outcomes were all-cause mortality, any venous thromboembolism (VTE), and adverse events (AEs) leading to treatment discontinuation. We used the random-effects model for primary analyses and conducted sensitivity analyses using the leave-one-out and fixed-effects models. Furthermore, certainty of evidence was assessed using the GRADE framework.

**Results:**

Twenty randomized comparisons (involving 11,418 participants) were included in this study. Roxadustat was associated with higher odds of VAT (odds ratio (OR): 1.50; 95% confidence interval (CI): 1.06–2.12) and all-cause mortality (OR: 1.14; 95% CI: 1.06–1.22) with minimal heterogeneity; it was also found to increase AEs leading to discontinuation (OR: 1.76; 95% CI: 1.48–2.10). For any VTE, the estimate was imprecise and had a wide CI including the null value (OR: 3.69; 95% CI: 0.71–19.14). Subgroup analyses showed no evidence of effect modification for mortality by dialysis status or comparator type. For VAT, the subgroup estimates were directionally adverse for both the dialysis-dependent (DD) and non-dialysis-dependent (NDD) populations. However, the primary clinical interpretation of VAT pertained to the DD population because the majority of NDD patients lacked established vascular access; thus, the NDD findings warrant cautious interpretation. Discontinuation due to AEs increased in both the DD and NDD trials, with larger effects in the DD and ESA-controlled trials. Certainty of evidence was moderate for mortality and VAT but low for any VTE and AE-related discontinuation.

**Conclusion:**

In this meta-analysis of anemia in CKD, roxadustat was found to be associated with higher odds of VAT and all-cause mortality, along with increased AEs leading to treatment discontinuation, whereas the effects on any VTE remained uncertain because of imprecise results. These findings support careful selection of patients, close surveillance of hemoglobin levels after treatment initiation or dose adjustment, and continued monitoring of vascular access when using roxadustat in dialysis settings.

## 1 Introduction

Anemia is a common and clinically consequential complication of chronic kidney disease (CKD) (Iatridi et al., 2025; Zheng et al., 2025), with a prevalence that increases as kidney function declines, exceeding 90% among patients receiving renal replacement therapy (Nakhoul et al., 2016). In CKD populations, anemia is associated with impaired functional status and poorer health-related quality of life. It is also linked to higher transfusion requirements and increased risks of hospitalization and death (Hörl, 2013; Hanna et al., 2021). Current management strategies for anemia primarily integrate iron therapy and erythropoiesis-stimulating agents (ESAs), while transfusion of red blood cells is reserved only for select clinical situations (Locatelli et al., 2013). However, randomized trials pursuing higher hemoglobin targets with ESAs have reported greater cardiovascular and thrombotic risks, informing more conservative treatment targets and sustained interest in alternative approaches (Besarab et al., 1998; Singh et al., 2006; Pfeffer et al., 2009; Palmer et al., 2010).

Roxadustat is an oral hypoxia-inducible factor prolyl hydroxylase inhibitor (HIF-PHI) developed to increase endogenous erythropoietin production, coordinate iron handling, and lower circulating hepcidin levels (Provenzano et al., 2016a; Besarab et al., 2015). Phase 2 studies in non-dialysis and dialysis populations of CKD have shown dose-dependent hemoglobin responses and suggested favorable effects on iron indices, thereby supporting longer-term evaluations (Provenzano et al., 2016a; 2016b; Chen et al., 2017). Subsequent phase 3 trials, which expanded exposure across non-dialysis and dialysis populations using placebo or ESA comparators, have confirmed hemoglobin efficacy over clinically relevant follow-up periods (Chen et al., 2019a; Charytan et al., 2021; Chen et al., 2019b; Fishbane et al., 2022; Provenzano et al., 2021a).

Although several meta-analyses have shown improvements in hemoglobin and iron indices with roxadustat (Liu et al., 2020; Abdelazeem et al., 2022; Tang et al., 2021), safety estimates, particularly regarding cardiovascular signals, have been less consistent across analyses and populations (Abdelazeem et al., 2022; Tang et al., 2021; Li et al., 2024). Thrombotic outcomes are particularly sensitive to endpoint definition and ascertainment; as these events are uncommon, they are often captured through serious adverse event (SAE) reporting using the preferred terms of the Medical Dictionary for Regulatory Activities (MedDRA) rather than adjudicated clinical endpoints (Chen et al., 2019a; Charytan et al., 2021; Fishbane et al., 2022). In dialysis-dependent (DD) CKD, vascular access thrombosis (VAT) is a clinically important concern as it can lead to missed dialysis, hospitalization, urgent intervention, and loss of access; therefore, accurate quantification is central to the benefit-risk assessment (Quencer et al., 2017; Roy-Chaudhury et al., 2003). Earlier pooled analyses of roxadustat and recent meta-analyses, including network meta-analyses, have generally evaluated broader cardiovascular or composite safety outcomes rather than VAT as the central clinical endpoint (Barratt et al., 2021a; Provenzano et al., 2021b; Ren et al., 2024).

Hypoxia and HIF activation intersect with thrombosis biology through their effects on coagulation programs and inflammatory thrombosis pathways, providing a biological rationale for scrutinizing thrombotic safety signals under sustained HIF pathway modulation (Evans, 2019). Vascular endothelial growth factor (VEGF) has also been linked to risk of venous thromboembolism (VTE) in genetic analysis, further supporting systematic evaluation of thrombotic outcomes with HIF-PHIs (Zhang et al., 2022). Against this background, we conducted a systematic review and meta-analysis of randomized trials comparing roxadustat with placebo or ESAs by designating VAT as the primary outcome to provide a more dialysis-relevant and clinically actionable assessment focused on access patency and treatment continuity. We also quantified all-cause mortality, any VTE, and treatment discontinuation, along with assessing the certainty of evidence using the Grading of Recommendations Assessment, Development, and Evaluation (GRADE) framework (Evans, 2019; Zhang et al., 2022; Guyatt et al., 2008).

## 2 Methods

### 2.1 Data sources and search strategy

Our systematic review was conducted in accordance with the Preferred Reporting Items for Systematic Reviews and Meta-Analyses (PRISMA) guidelines and was registered with PROSPERO (registration ID: CRD420251274247; available at https://www.crd.york.ac.uk/prospero/display_record.php?ID=CRD420251274247). The eligibility criteria and outcomes were specified in the registered PROSPERO record, while the relevant protocol details and post-registration clarifications are summarized in Supplementary Table S2. We searched records regarding roxadustat in PubMed, Embase, Web of Science, and the Cochrane Library from database inception up to 21 August 2025. The search strategy combined database-specific controlled vocabulary (e.g., MeSH and Emtree) with free-text terms for roxadustat and its development code (FG-4592), along with terms related to anemia, CKD, dialysis, and randomized controlled trials (RCTs). The complete database-specific search strategy is outlined in Supplementary Table S3. We also manually screened the reference lists of relevant reviews and eligible reports to identify additional studies.

### 2.2 Eligibility criteria

The eligibility criteria were defined *a priori* using the PICOS framework. We included parallel-group RCTs that enrolled adults (≥18 years) with CKD comprising dialysis-dependent (DD) and non-dialysis-dependent (NDD) populations. The eligible studies were required to randomize participants to roxadustat and a clearly defined control group. The comparators included a placebo (with permission for protocol-specified ESA rescue) or active ESAs (e.g., epoetin alfa or darbepoetin alfa). The included trials were required to report extractable data for at least one pre-specified outcome. The primary outcome of our analysis was VAT, while the secondary outcomes included all-cause mortality, any VTE, and adverse events (AEs) leading to discontinuation of the study treatment.

The data on VAT and any VTE were derived from SAE tables based on MedDRA preferred terms. VAT was defined using terms related to arteriovenous fistula (AVF) and/or arteriovenous graft (AVG) thrombosis. When both categories were reported in a trial, the event counts were summed; if only one category was used, then its values were used as is. Similarly, VTE was derived as a composite of deep-vein thrombosis (DVT) and pulmonary embolism (PE). We acknowledge that summing up the counts of the preferred terms may overestimate participant-level incidence because of potential lack of mutual exclusivity (i.e., double counting); however, this approach was used to maximize event capture, given the incomplete reporting of composite endpoints. We excluded non-randomized or single-arm studies, pediatric populations, studies of non-CKD indications, and reports in which data could not be isolated for roxadustat-specific randomized comparisons.

### 2.3 Selection process and data extraction

The study selection and data extraction were performed independently by two investigators (JT and YZe). Screening was conducted in two stages, namely an initial review of titles and abstracts, followed by a full-text assessment of potentially eligible reports. The data were extracted using a pre-specified standardized extraction template. Disagreements were resolved through discussion to achieve consensus, and a third investigator (XL) served as an adjudicator when necessary.

### 2.4 Risk of bias assessment

Two independent reviewers (JT and YZe) assessed the risk of bias for the included RCTs using the revised Cochrane Risk of Bias tool. This assessment covered five core domains as follows: (1) randomization process; (2) deviations from intended interventions; (3) missing outcome data; (4) measurements of the outcomes; and (5) selection of the reported results. Discrepancies between the reviewers were resolved through discussion or adjudication by a third investigator.

### 2.5 Data analysis and sensitivity assessment

The data analyses were conducted within a frequentist framework using the “meta” package in R (version 4.3.2). Dichotomous outcomes were summarized in terms of odds ratios (ORs) with 95% confidence intervals (CIs). We used the random effects model as the primary approach to estimate between-study variance (τ^2^) via the restricted maximum likelihood method and calculated the CIs using the Hartung–Knapp–Sidik–Jonkman method (IntHout et al., 2014). Heterogeneity was quantified using I^2^ and τ^2^, and the 95% CIs were reported to estimate the expected range of effects in future settings (Higgins et al., 2009). When handling sparse data, a continuity correction of 0.5 was applied only to studies that reported zero events in a single arm. Trials with zero events in both arms were excluded from the primary pooling of Ors, as they provide no information on the relative effect magnitude in this framework (Sweeting et al., 2004; Friedrich et al., 2007). Sensitivity analyses were conducted using the fixed effects (common effects) model and by assessing the influence of individual studies using the leave-one-out approach.

Subgroup analyses were pre-specified by dialysis status (DD vs. NDD) and comparator type (placebo vs. ESA), and the differences were assessed using the χ^2^ statistic. The dialysis status analysis included only trials enrolling exclusively DD or NDD populations, or those reporting stratified data. Similarly, the comparator analysis was restricted to trials with a single randomized control group (placebo or ESA). Trials reporting more than one independent randomized cohort were extracted and analyzed as separate comparisons. Publication bias was assessed by visually inspecting the funnel plots and using Egger’s regression test for outcomes with ten or more studies (Egger et al., 1997; Sterne et al., 2011).

### 2.6 Evidence certainty assessment

The certainty of evidence was assessed using the GRADE framework. Here, two independent reviewers (JT and YZe) evaluated each outcome against the five core domains of risk of bias, inconsistency, indirectness, imprecision, and publication bias. Evidence derived from the randomized trials was initially classified as high certainty and was downgraded by one or two levels based on the presence of serious or very serious limitations in any domain. Discrepancies were resolved through discussion or adjudication by a third investigator (XL).

## 3 Results

### 3.1 Study selection and characteristics

**FIGURE 1:**
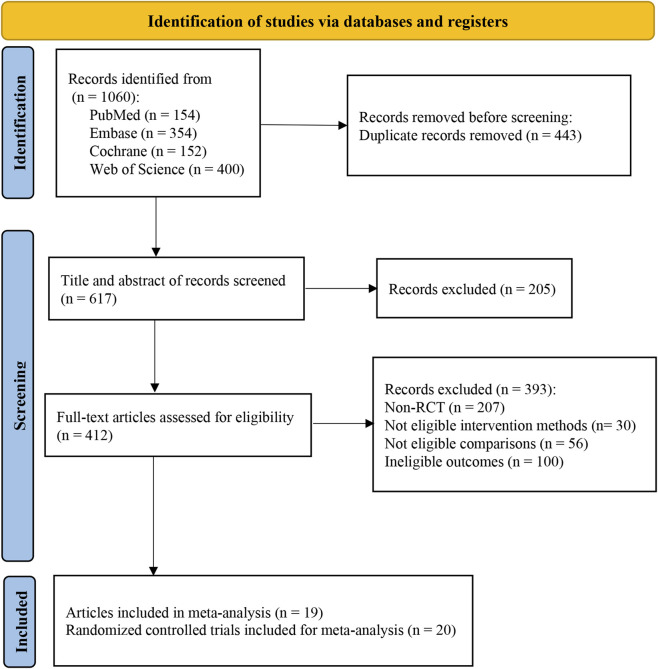
PRISMA flow diagram of the study selection process.

A total of 19 trial reports comprising 20 randomized comparisons involving 11,418 participants were included in the meta-analysis (Figure 1) (Besarab et al., 2015; Provenzano et al., 2016b; Chen et al., 2017; Chen et al., 2019a; Charytan et al., 2021; Chen et al., 2019b; Fishbane et al., 2022; Provenzano et al., 2021a; Fishbane et al., 2021; Shutov et al., 2021; Barratt et al., 2021b; Coyne et al., 2021; Akizawa et al., 2021; Akizawa et al., 2019; Csiky et al., 2021; Akizawa et al., 2020; Hou et al., 2022; Tan et al., 2024; Wu et al., 2024). The detailed study characteristics are summarized in Table 1. One publication reported two independent randomized cohorts, including an NDD placebo-controlled cohort and a DD ESA-controlled cohort (Chen et al., 2017).

**TABLE 1:** Baseline characteristics of the included studies.

Author, year	Trial ID	Country	Mean age (years)	Female% (Rox/Ctrl)	Hb (g/dL)	Dialysis status	Intervention (starting dose)	Comparator	n (Rox)	n (Ctrl)	Follow-up duration
Fishbane et al. (2021)	NCT02174627	Global	62.4	58/58	9.1	NDD	70 mg TIW	Placebo	1393	1388	16.4 months
Shutov et al. (2021)	NCT01887600	Europe	63.8	56/55	9.1	NDD	Weight-based TIW	Placebo	278	278	104 weeks
Barratt et al. (2021b)	NCT02021318	Europe	66.5	54/53	9.5	NDD	Weight-based TIW	Darbepoetin alfa	323	293	104 weeks
Coyne et al. (2021)	NCT01750190	Global	64	58/59	9.1	NDD	Weight-based TIW	Placebo	616	305	104 weeks
Chen et al. (2019b)	NCT02652819	China	58	48/36	8.9	NDD	Weight-based TIW	Placebo	101	50	26 weeks
Akizawa et al. (2021)	NCT02988973	Japan	68.9	48/46	10.3	NDD	50/70/100 mg TIW	Darbepoetin alfa	132	131	52 weeks
Besarab et al. (2015)	NCT00761657	USA	62	66/54	9.7	NDD	0.7–2.0 mg/kg TIW	Placebo	88	28	4 weeks
Akizawa et al. (2019)	NCT01964196	Japan	67.5	40/37	9.8	NDD	50/70/100 mg TIW	Placebo	80	27	24 weeks
Chen et al. (2017)	NCT01599507	China	51.4	42/45	8.8	NDD	1.1–2.25 mg/kg TIW	Placebo	61	30	8 weeks
Fishbane et al. (2022)	NCT02174731	Global	54.8	43/42	9.6	DD (HD)	70–100 mg or ESA-based	Epoetin alfa	1068	1065	104 weeks
Provenzano et al. (2021a)	NCT02052310	Global	56	44/45	8.3	DD (Inc)	70–100 mg TIW	Epoetin alfa	522	517	104 weeks
Charytan et al. (2021)	NCT02273726	USA/Global	55.7	45/48	9.6	DD (HD)	ESA-based TIW	Epoetin alfa	370	370	104 weeks
Csiky et al. (2021)	NCT02964936	Europe	62.5	40/37	9.7	DD	ESA-based TIW	ESA (Var)	415	421	104 weeks
Chen et al. (2019a)	NCT02652806	China	49	38/43	8.7	DD	100/120 mg TIW	Epoetin alfa	204	101	26 weeks
Akizawa et al. (2020)	NCT02952092	Japan	61.6	32/37	10.9	DD (HD)	70/100 mg TIW	Darbepoetin alfa	150	152	24 weeks
Hou et al. (2022)	ChiCTR2000035054	China	53	42/53	8	DD (PD)	100/120 mg TIW	ESA	86	43	24 weeks
Tan et al. (2024)	ChiCTR2000041202	China	51.2	40/49	10.5	DD (HD)	Titrated TIW	rHuEPO	57	57	24 weeks
Wu et al. (2024)	NCT04655027	China	55.1	30/41	8.7	DD/NDD	Standard TIW	rHuEPO	13	12	2 weeks
Provenzano et al. (2016b)	NCT01147666	USA	53.8	45/39	10.2	DD	1.0–2.0 mg/kg TIW	Epoetin alfa	83	23	19 weeks
Chen et al. (2017)	NCT01596855	China	51	35/41	10.7	DD	1.1–2.3 mg/kg TIW	Epoetin alfa	65	22	6 weeks

Values are presented as the mean or percentage. Abbreviations: Rox, roxadustat; Ctrl, control; DD, dialysis-dependent; ESA, erythropoiesis-stimulating agent; Hb, hemoglobin; HD, hemodialysis; Inc, incident dialysis; NDD, non-dialysis-dependent; PD, peritoneal dialysis; rHuEPO, recombinant human erythropoietin; TIW, three times weekly; Var, variable ESA type (epoetin alfa or darbepoetin alfa).

### 3.2 Risk of bias

**FIGURE 2:**
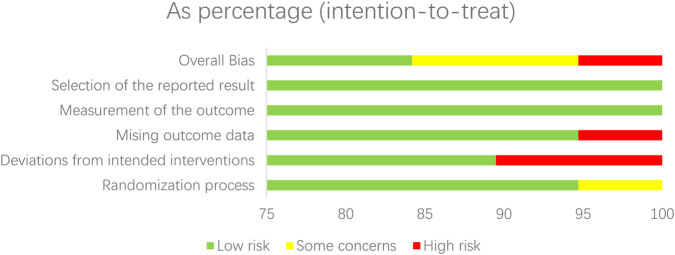
Assessment of bias for inclusion as a randomized controlled trial in the meta-analysis.

The risk of bias assessments are summarized in Figure 2. The majority of domains across the included trials were judged as low risk. High-risk designations were concentrated in domains that deviated from the intended interventions or had missing outcome data, whereas ratings of “some concerns” were primarily attributable to the randomization process. Overall, the majority of comparisons were classified as low risk, while a few subsets exhibited some concerns or were classified as high risk.

### 3.3 Primary outcome

**FIGURE 3:**
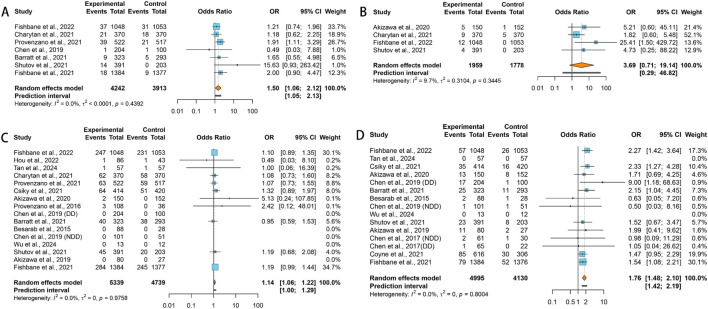
Forest plots of the thrombotic outcomes, mortality, and discontinuation for trials involving anemia in chronic kidney disease (CKD) that compared roxadustat with a control group: **(A)** vascular access thrombosis (VAT); **(B)** any venous thromboembolism (VTE); **(C)** all-cause mortality; and **(D)** adverse events (AEs) leading to treatment discontinuation.

The primary analysis of VAT included seven trials (Figure 3A); here, roxadustat was associated with significantly higher odds of VAT than the controls (OR: 1.50; 95% CI: 1.06–2.12), with no evidence of statistical heterogeneity (I^2^ = 0.0%, τ^2^ < 0.0001). Notably, the 95% CI (1.05–2.13) excluded unity, suggesting that this increased risk is likely to be a consistent finding across comparable future study populations. The pooled estimates remained stable in the leave-one-out analyses (Supplementary Figure S1A), and the fixed effects modeling yielded directionally consistent results (Supplementary Figure S2A). The certainty of evidence for VAT was graded as moderate (Table 2).

**TABLE 2:** GRADE evidence profiles for the overall quality of the evidence assessment.

Certainty assessment							No. of patients		Effects		Certainty	Importance
№ of studies	Study design	Risk of bias	Inconsistency	Indirectness	Imprecision	Other considerations	[Intervention]	[Comparison]	Relative (95% CI)	Absolute (95% CI)		
All-cause mortality												
16	Randomized trials	Serious^a^	Not serious	Not serious	Not serious	None	5,339/10,078 (53.0%)	4,739/10,078 (47.0%)	OR: 1.14 (1.06–1.22)	10 more per 1,000 (from 4 more to 16 more)^b^	⊕⊕⊕○ Moderate^a^	CRITICAL
VAT												
7	Randomized trials	Serious^c^	Not serious	Not serious	Not serious	None	4,242/8,155 (52.0%)	3,913/8,155 (48.0%)	OR: 1.50 (1.06–2.12)	33 more per 1,000 (from 4 more to 73 more)^d^	⊕⊕⊕○ Moderate^c^	CRITICAL
AEs leading to treatment discontinuation												
15	Randomized trials	Very serious^e^	Not serious	Not serious	Not serious	None	4,995/9,125 (54.7%)	4,130/9,125 (45.3%)	OR: 1.76 (1.48–2.10)	36 more per 1,000 (from 23 more to 52 more)^f^	⊕⊕○○ Low^e^	CRITICAL
Any VTE												
4	Randomized trials	Serious^g^	Not serious	Not serious	Serious^h^	None	1,959/3,737 (52.4%)	1,778/3,737 (47.6%)	OR: 3.69 (0.71–19.14)	16 more per 1,000 (from 2 fewer to 100 more)^i^	⊕⊕○○ Low^g,h^	CRITICAL

Abbreviations: VAT, vascular access thrombosis; CI, confidence interval; OR, odds ratio; AE, adverse event; VTE, venous thromboembolism.

### 3.4 Secondary outcomes

Data for the composite endpoints of any VTE were available from four trials (Figure 3B). The pooled estimates suggested increased odds with roxadustat but lacked precision (OR: 3.69; 95% CI: 0.71–19.14) and were accompanied by low heterogeneity (I^2^ = 9.7%, τ^2^ = 0.3104) (Figure 3B). The 95% CI was wide (0.29–46.82) and included unity, reflecting sparse events and substantial uncertainty regarding effect magnitudes. Although the fixed effects sensitivity analysis yielded a statistically significant estimate (OR: 4.22; 95% CI: 1.84–9.67; Supplementary Figure S2B), this result was heavily driven by a single large trial (∼70% weight). Consequently, given the sparse event counts, the primary inference was anchored upon the more conservative random effects model. The leave-one-out estimates of the ORs ranged from 2.44 to 7.84 (Supplementary Figure S1B), and the certainty of evidence was rated as low (Table 2).

In the pooled analysis of 16 trials, roxadustat was associated with significantly higher odds of all-cause mortality than the controls (OR: 1.14; 95% CI: 1.06–1.22), with negligible between-study heterogeneity (I^2^ = 0.0%, τ^2^ = 0) (Figure 3C). The 95% CI (1.00–1.29) indicates that the effects in comparable future trials may likely range from the null effect to a moderate increase in mortality risk. These results remained robust in the leave-one-out analyses (OR: 1.11–1.16; Supplementary Figure S1C) and in fixed effects modeling (OR: 1.14; 95% CI: 1.02–1.28; Supplementary Figure S3A). The certainty of evidence for mortality was graded as moderate (Table 2); the funnel plots were broadly symmetric, and Egger’s test indicated no evidence of small-study effects (*p* = 0.723; Supplementary Figure S4). Regarding safety-related attrition, roxadustat increased the odds of treatment discontinuation due to AEs across 15 trials (OR: 1.76; 95% CI: 1.48–2.10; I^2^ = 0.0%) (Figure 3D). The 95% CI (1.42–2.19) excluded unity, supporting the consistency of this risk across similar settings. Sensitivity analyses using leave-one-out (OR: 1.67–1.87; Supplementary Figure S1D) and fixed effects modeling (OR: 1.80; 95% CI: 1.48–2.19; Supplementary Figure S3B) confirmed the stability of this finding. The certainty of evidence was rated as low (Table 2); inspection of the funnel plots revealed no obvious asymmetries, and Egger’s test was not significant (*p* = 0.891; Supplementary Figure S5).

Evidence certainty according to the GRADE framework: High certainty: We are very confident that the true effects are close to the estimated effects. Moderate certainty: We are moderately confident in the effects estimate; the true effects are likely to be close to the estimated effects, but there is a possibility that they may be substantially different. Low certainty: Our confidence in the effects estimate is limited; the true effects may be substantially different from the estimated effects. Very low certainty: We have very little confidence in the effects estimate.

Explanations of the superscripts in Table 2:

- Downgraded by one level for risk of bias: The pooled estimate is influenced by open-label trials; although mortality is an objective endpoint, the lack of blinding introduces some concerns regarding deviations from the intended interventions.
- Calculated based on an assumed control risk (ACR) of 80 per 1,000 (median event rate in the control groups).
- Downgraded by one level for risk of bias: The analysis includes open-label trials, and the lack of blinding may influence the reporting or detection of thrombotic events related to vascular access.
- Calculated based on an ACR of 70 per 1,000 (median event rate in the control groups).
- Downgraded by two levels for very serious risk of bias: The outcome (discontinuation) was subjective and highly susceptible to performance bias in open-label designs; knowledge of treatment assignment likely influenced the decision to discontinue treatment.
- Calculated based on an ACR of 50 per 1,000 (median event rate derived from the control groups).
- Downgraded by one level for risk of bias: The trials were predominantly open-label designs.
- Downgraded by one level due to serious imprecision: The 95% CI was very wide (0.71–19.14) and included the line of no effect (1.0), consistent with both appreciable benefit and appreciable harm; additionally, the analysis was limited by sparse data (low number of total events).
- Calculated based on an ACR of 6 per 1,000 (median event rate derived from the control groups).

Note on absolute effects: The absolute risk difference and its 95% CI were calculated based on the ACR derived from the median event rate in the control groups (approximately 80/1,000 for all-cause mortality, 70/1,000 for VAT, 50/1,000 for AEs leading to treatment discontinuation, and 6/1,000 for any VTE).

### 3.5 Subgroup analysis

**FIGURE 4:**
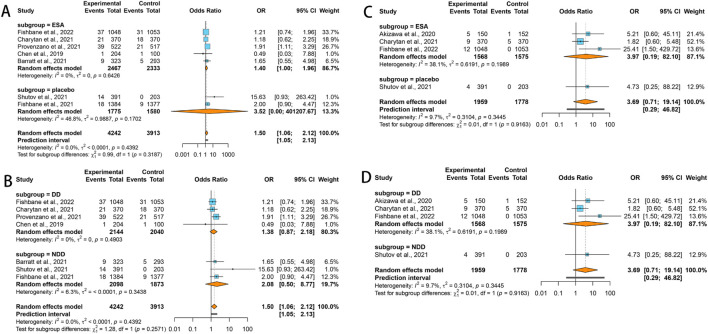
Subgroup forest plots for VAT and any VTE: **(A)** VAT by comparator type (erythropoiesis-stimulating agent (ESA) vs. placebo); **(B)** VAT by dialysis status (dialysis-dependent (DD) vs. non-dialysis-dependent (NDD)); **(C)** any VTE by comparator type (ESA vs. placebo); **(D)** any VTE by dialysis status (DD vs. NDD). The VAT findings in the NDD subgroup should be interpreted cautiously because the majority of NDD patients lack established vascular access, which limits the clinical interpretability of this population relative to the DD subgroup.

**FIGURE 5:**
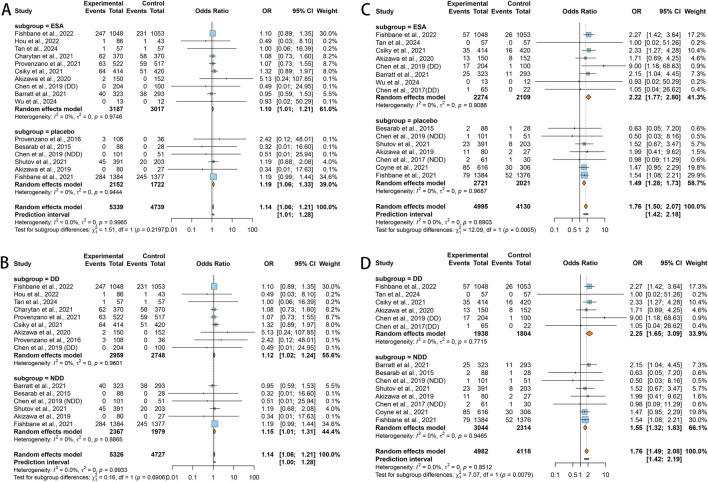
Subgroup forest plots for all-cause mortality and AEs leading to treatment discontinuation: **(A)** all-cause mortality by comparator type (ESA vs. placebo); **(B)** all-cause mortality by dialysis status (DD vs. NDD); **(C)** discontinuation due to AEs by comparator type (ESA vs. placebo); **(D)** discontinuation due to AEs by dialysis status (DD vs. NDD).

The results of the subgroup analyses, stratified by comparator type (ESA vs. placebo) and dialysis status (DD vs. NDD), are summarized in Figures 4, 5. Regarding VAT, roxadustat was associated with a borderline significant increase in odds in the ESA-controlled stratum (OR: 1.40; 95% CI: 1.00–1.96; I^2^ = 0.0%); in contrast, the placebo-controlled estimate was statistically unstable owing to zero-event arms (OR: 3.52; 95% CI: 0.00–401,207.67), which rendered the CI uninformative. No significant modifications were detected based on comparator type (*p* = 0.3187). When stratified by dialysis status, the VAT estimates were directionally adverse in both the DD and NDD populations, and the formal interaction test was not statistically significant (*p* = 0.2571). However, this should not be interpreted as evidence of equivalent clinical risk across the two populations. Because the majority of NDD patients lack established vascular access, their anatomical predispositions to VAT differ fundamentally from those of the DD patients, which render the VAT findings in the NDD stratum less readily clinically interpretable. Hence, the primary clinical interpretation of VAT in this study rests with the DD population, whereas the NDD findings should be interpreted cautiously and considered exploratory.

For the VTE composite, the estimates remained imprecise across all strata. The ESA-controlled subgroup showed a numerically higher but highly imprecise estimate (OR: 3.97; 95% CI: 0.19–82.10), while the placebo subgroup relied on a single study with sparse events. No evidence of effect modification was observed for either comparator type (*p =* 0.9163) or dialysis status (*p =* 0.9163). In terms of all-cause mortality, the risk increase with roxadustat was consistent across the comparator-based and dialysis-based subgroups. Significant increases were observed versus both ESAs (OR: 1.10; 95% CI: 1.01–1.21) and placebo (OR: 1.19; 95% CI: 1.06–1.33), and there were no significant differences between the comparator-based subgroups (*p =* 0.2197). Similarly, results were consistent across dialysis settings [DD (OR: 1.12, 95% CI: 1.02–1.24); NDD (OR: 1.15, 95% CI: 1.01–1.31)], with no significant differences between the subgroups (*p* = 0.6906).

Conversely, a significant quantitative interaction was identified for discontinuation due to AEs. Although roxadustat increased the discontinuation rates in all subgroups, the magnitude of effects was more pronounced in ESA-controlled trials (OR: 2.22; 95% CI: 1.77–2.80) than in the placebo-controlled trials (OR: 1.49; 95% CI: 1.28–1.73; *p =* 0.0005). This pattern was also observed in the dialysis stratification [DD (OR: 2.25, 95% CI: 1.65–3.09) vs. NDD (OR: 1.55, 95% CI: 1.32–1.83); *p =* 0.0079]. It is notable that the comparator types and dialysis statuses overlapped substantially in the included trials (e.g., the majority of DD trials used ESAs), suggesting that these findings likely reflect correlated study design features rather than independent effect modifiers.

## 4 Discussion

Our meta-analysis of 19 RCTs comprising 20 randomized comparisons and involving 11,418 participants showed that roxadustat was associated with significantly increased risks of VAT and all-cause mortality in patients with CKD. In addition, discontinuation due to AEs occurred more frequently with roxadustat than with the placebo or ESA comparators. Compared with previous evidence syntheses that emphasized broader efficacy and safety outcomes, including cardiovascular events, our study positions VAT as the primary endpoint (Liu et al., 2020; Abdelazeem et al., 2022; Tang et al., 2021). This distinction is clinically relevant because VAT is not merely another thrombotic complication; in dialysis practice, it can compromise access patency, disrupt treatment continuity, and necessitate urgent access intervention (Quencer et al., 2017; Roy-Chaudhury et al., 2003). By framing VAT as the primary outcome, the present analysis provides a more clinically actionable perspective on the safety profile of roxadustat in CKD anemia.

The VAT signals identified here are clinically important for the DD population. The pooled estimate indicated a 50% increase in odds (OR: 1.50) with negligible between-study heterogeneity. In hemodialysis, VAT is a critical complication that can precipitate missed treatments, urgent interventions, and hospitalization, ultimately jeopardizing long-term access viability (Quencer et al., 2017). Because vascular access dysfunction is a major source of morbidity, this signal warrants careful patient selection (Roy-Chaudhury et al., 2003). In patients with a history of recurrent thrombosis or limited access options, initiation of roxadustat treatment warrants particular caution. Rigorous access surveillance is also advised after transitioning to this therapy. An important interpretive caveat here is that the results of subgroup analysis by dialysis status should not be overread (Sun et al., 2014). Although the formal interaction test did not show a statistically significant subgroup difference, the DD and NDD populations differed fundamentally in their vascular access background. Because the majority of NDD patients lacked mature AVFs or AVGs, the VAT findings in this subgroup were less readily clinically interpretable. Thus, the main clinical inference regarding VAT rests with the DD population, whereas the NDD findings should be interpreted cautiously and considered exploratory rather than as evidence of risk equivalence across strata (Sun et al., 2014).

Regarding survival outcomes, roxadustat was associated with a modest but statistically significant increase in all-cause mortality compared to the controls. This finding is of particular concern in CKD, where the cardiovascular burden is already substantial (Go et al., 2004; Sarnak et al., 2003). Historically, drug trials targeting higher hemoglobin levels with ESAs have failed to improve the outcomes and have also increased cardiovascular or thrombotic events in several instances (Besarab et al., 1998; Singh et al., 2006; Pfeffer et al., 2009; Palmer et al., 2010). These findings reinforce the need to weigh the potential benefits of hemoglobin correction and transfusion avoidance against the observed increase in mortality risk, particularly in patients with limited physiological reserves or advanced cardiovascular disease. A practical implication of these findings is the need for close hemoglobin surveillance and careful dose titration during roxadustat therapy. The KDIGO guideline recommends checking the hemoglobin level 2–4 weeks after initiation of HIF-PHI therapy or dose adjustment and then at approximately 4-week intervals during treatment (Kidney Disease: Improving Global Outcomes Anemia Work Group, 2026). Recent observational data have also described hemoglobin overshoots after switching from ESAs to roxadustat (Tamaki et al., 2025). Although these observations do not establish a causal explanation for the thrombotic signals observed herein, they support careful monitoring during the early stages of treatment to avoid abrupt hemoglobin excursions and target level overshoots, particularly in patients with vascular access or other thrombotic susceptibilities.

Tolerability was also found to be an important differentiating factor. Roxadustat increased the odds of discontinuation due to AEs by approximately 76% (OR: 1.76). This attrition is clinically relevant because unplanned cessation can destabilize anemia management and necessitate rescue therapies, such as transfusions or ESA switching, thereby introducing additional risks. This finding underscores the importance of monitoring for early intolerance to support treatment persistence. In contrast to the clear signals for VAT, the risk of any VTE remained statistically indeterminate. The wide CIs observed in our analysis likely reflect the sparse events and limitations in primary outcome ascertainment. In many phase 3 trials, the thrombotic events were captured using broad MedDRA AE/SAE codes rather than adjudicated VTE endpoints (Chen et al., 2019a; Fishbane et al., 2022). Because these patients may have been listed under multiple preferred terms, the participant-level incidence rate cannot be derived reliably by simply combining the DVT and PE counts. In addition, the inconsistent handling of zero-event trials across prior meta-analyses may have contributed to the conflicting safety conclusions reported in the literature (Abdelazeem et al., 2022; Tang et al., 2021; Li et al., 2024). Given these assessment limitations, the moderate certainty assigned to VAT and mortality contrasts with the low certainty for any VTE, suggesting that venous outcomes should be interpreted cautiously (Guyatt et al., 2008).

From a mechanistic perspective, the concern regarding sustained HIF pathway modulation shifting the hemostatic balance remains. Experimental models suggest that hypoxic environments can induce a pro-thrombotic phenotype in the vascular wall (Matsuura et al., 2015) and suppress fibrinolysis via plasminogen activator inhibitor-1 upregulation (Pinsky et al., 1998). Additionally, pathways upstream of HIF-1α have been implicated in the regulation of neutrophil extracellular trap formation (McInturff et al., 2012), and Mendelian randomization studies have linked elevated VEGF levels to VTE risk (Zhang et al., 2022). Although mechanistic plausibility does not establish clinical risk, these pathways are directionally consistent with the thrombotic signals observed in our trial-level analysis. The broader therapeutic landscape of HIF-PHIs continues to evolve across jurisdictions. In addition to roxadustat, other agents in this class include daprodustat, vadadustat, enarodustat, desidustat, and molidustat. In the European Union, roxadustat has been authorized for the treatment of symptomatic anemia associated with CKD, whereas vadadustat has been authorized for the treatment of adults on chronic maintenance dialysis. In the United States, daprodustat has been approved for adults receiving dialysis. In Asian countries, the regulatory landscape is more heterogeneous; enarodustat has been cleared for clinical use in Japan, and desidustat has been approved in India for the treatment of anemia in patients with CKD. These regional differences in regulatory scope and target populations underscore the fact that the present findings should not be extrapolated uncritically to HIF-PHIs as a class (Kidney Disease: Improving Global Outcomes Anemia Work Group, 2026; Stoumpos et al., 2024).

Several limitations of the included trials and our analysis merit consideration. First, although follow-up efforts were generally balanced between treatment arms within individual trials, the study durations varied substantially across the included RCTs and ranged from a few weeks to 104 weeks. Pooling the dichotomous event counts across trials with such disparate follow-up periods may not fully capture exposure-adjusted risks for time-dependent outcomes, such as VAT and all-cause mortality. Because the hazard ratios and exposure-adjusted incidence rates were not consistently available across eligible trials, our analysis relied on cumulative event counts. Second, the VAT and VTE outcomes were derived from the MedDRA preferred terms reported in the SAE tables rather than from uniformly adjudicated endpoints, which may have affected the event ascertainment. Third, a substantial portion of the included trials were open-label studies, which could have influenced some of the safety outcomes, particularly treatment discontinuation. Future meta-analyses using time-to-event or exposure-adjusted measures, along with more standardized endpoint adjudication, may provide more refined estimates of risk.

Collectively, the above findings suggest the need to refine clinical strategies for managing anemia in CKD. Prudent practices should emphasize rigorous risk stratification before treatment initiation. In the hemodialysis population, the specific VAT signal warrants intensified vascular access monitoring with a prompt assessment upon emergence of early signs of access dysfunction (Quencer et al., 2017; Roy-Chaudhury et al., 2003). Furthermore, given the substantial background burden of cardiovascular-related mortality in CKD, a conservative approach is advisable for patients with established cardiovascular diseases or a history of thrombosis (Go et al., 2004; Sarnak et al., 2003). Ultimately, the therapeutic decisions should contextualize the convenience of oral administration against these safety signals; should the benefit-risk profile appear unfavorable, transitioning to an established alternative (such as iron optimization, ESAs, or transfusion) remains a sound management strategy.

## 5 Conclusion

Roxadustat was found to be associated with an increased risk of all-cause mortality and VAT compared to treatment with placebo or ESAs, as supported by the moderate-certainty evidence. Roxadustat was also found to be associated with higher rates of discontinuation due to AEs, and these safety signals were robust across the sensitivity analyses. The clinical interpretation of VAT is most directly relevant to DD populations with established vascular access. These findings support careful selection of patients, close surveillance of hemoglobin levels after treatment initiation or dose adjustment, continuous monitoring of vascular access in dialysis settings, and early assessment of treatment tolerability when using roxadustat.

## Data Availability

The datasets presented in this study can be found in online repositories. The names of the repositories and accession numbers can be found in the article/Supplementary Material.
