# Supplementary material for: Thrombotic outcomes and mortality with roxadustat for anemia in chronic kidney disease: a systematic review and meta-analysis of randomized trials
Source: Front Pharmacol. 2026 Jun 10;17:1792709. doi: 10.3389/fphar.2026.1792709 (PMC13290462; doi:10.3389/fphar.2026.1792709)
Supplement: Supplementary file 1 [file DataSheet1.docx]

**Table S1 The PRISMA 2020 Checklist**

| **Section and Topic** | **Item #** | **Checklist item** | **Location where item is reported** |
| --- | --- | --- | --- |
| **TITLE** | | |  |
| Title | 1 | Identify the report as a systematic review. | Title page |
| **ABSTRACT** | | |  |
| Abstract | 2 | See the PRISMA 2020 for Abstracts checklist. | Abstract |
| **INTRODUCTION** | | |  |
| Rationale | 3 | Describe the rationale for the review in the context of existing knowledge. | 1. Introduction |
| Objectives | 4 | Provide an explicit statement of the objective(s) or question(s) the review addresses. | 1. Introduction |
| **METHODS** | | |  |
| Eligibility criteria | 5 | Specify the inclusion and exclusion criteria for the review and how studies were grouped for the syntheses. | 2.2 Eligibility criteria |
| Information sources | 6 | Specify all databases, registers, websites, organisations, reference lists and other sources searched or consulted to identify studies. Specify the date when each source was last searched or consulted. | 2.1 Data sources and search strategy |
| Search strategy | 7 | Present the full search strategies for all databases, registers and websites, including any filters and limits used. | Supplementary Table S3 |
| Selection process | 8 | Specify the methods used to decide whether a study met the inclusion criteria of the review, including how many reviewers screened each record and each report retrieved, whether they worked independently, and if applicable, details of automation tools used in the process. | 2.3 Selection process and data extraction |
| Data collection process | 9 | Specify the methods used to collect data from reports, including how many reviewers collected data from each report, whether they worked independently, any processes for obtaining or confirming data from study investigators, and if applicable, details of automation tools used in the process. | 2.3 Selection process and data extraction |
| Data items | 10a | List and define all outcomes for which data were sought. Specify whether all results that were compatible with each outcome domain in each study were sought (e.g. for all measures, time points, analyses), and if not, the methods used to decide which results to collect. | 2.2 Eligibility criteria; 2.3 Selection process and data extraction |
|  | 10b | List and define all other variables for which data were sought (e.g. participant and intervention characteristics, funding sources). Describe any assumptions made about any missing or unclear information. | 2.2 Eligibility criteria; 2.3 Selection process and data extraction |
| Study risk of bias assessment | 11 | Specify the methods used to assess risk of bias in the included studies, including details of the tool(s) used, how many reviewers assessed each study and whether they worked independently, and if applicable, details of automation tools used in the process. | 2.4 Risk of bias assessment |
| Effect measures | 12 | Specify for each outcome the effect measure(s) (e.g. risk ratio, mean difference) used in the synthesis or presentation of results. | 2.5 Data analysis and sensitivity assessment |
| Synthesis methods | 13a | Describe the processes used to decide which studies were eligible for each synthesis (e.g. tabulating the study intervention characteristics and comparing against the planned groups for each synthesis (item #5)). | 2.5 Data analysis and sensitivity assessment |
|  | 13b | Describe any methods required to prepare the data for presentation or synthesis, such as handling of missing summary statistics, or data conversions. | 2.5 Data analysis and sensitivity assessment |
|  | 13c | Describe any methods used to tabulate or visually display results of individual studies and syntheses. | 2.5 Data analysis and sensitivity assessment |
|  | 13d | Describe any methods used to synthesize results and provide a rationale for the choice(s). If meta-analysis was performed, describe the model(s), method(s) to identify the presence and extent of statistical heterogeneity, and software package(s) used. | 2.5 Data analysis and sensitivity assessment |
|  | 13e | Describe any methods used to explore possible causes of heterogeneity among study results (e.g. subgroup analysis, meta-regression). | 2.5 Data analysis and sensitivity assessment |
|  | 13f | Describe any sensitivity analyses conducted to assess robustness of the synthesized results. | 2.5 Data analysis and sensitivity assessment |
| Reporting bias assessment | 14 | Describe any methods used to assess risk of bias due to missing results in a synthesis (arising from reporting biases). | 2.5 Data analysis and sensitivity assessment |
| Certainty assessment | 15 | Describe any methods used to assess certainty (or confidence) in the body of evidence for an outcome. | 2.6 Evidence Certainty Assessment |
| **RESULTS** | | |  |
| Study selection | 16a | Describe the results of the search and selection process, from the number of records identified in the search to the number of studies included in the review, ideally using a flow diagram. | Figure 1 |
|  | 16b | Cite studies that might appear to meet the inclusion criteria, but which were excluded, and explain why they were excluded. | Figure 1 |
| Study characteristics | 17 | Cite each included study and present its characteristics. | Table 1 |
| Risk of bias in studies | 18 | Present assessments of risk of bias for each included study. | 3.2 Risk of bias & Figure 2 |
| Results of individual studies | 19 | For all outcomes, present, for each study: (a) summary statistics for each group (where appropriate) and (b) an effect estimate and its precision (e.g. confidence/credible interval), ideally using structured tables or plots. | Figure 3 |
| Results of syntheses | 20a | For each synthesis, briefly summarise the characteristics and risk of bias among contributing studies. | 3.2 Risk of bias |
|  | 20b | Present results of all statistical syntheses conducted. If meta-analysis was done, present for each the summary estimate and its precision (e.g. confidence/credible interval) and measures of statistical heterogeneity. If comparing groups, describe the direction of the effect. | 3.3 Primary outcome & 3.4 Secondary outcomes |
|  | 20c | Present results of all investigations of possible causes of heterogeneity among study results. | 3.5 Subgroup analysis |
|  | 20d | Present results of all sensitivity analyses conducted to assess the robustness of the synthesized results. | 3.3 Primary outcome & Supplementary Figure S1, S2, S3 |
| Reporting biases | 21 | Present assessments of risk of bias due to missing results (arising from reporting biases) for each synthesis assessed. | 3.4 Secondary outcomes & Supplementary Figure S4, S5 |
| Certainty of evidence | 22 | Present assessments of certainty (or confidence) in the body of evidence for each outcome assessed. | Table 2 |
| **DISCUSSION** | | |  |
| Discussion | 23a | Provide a general interpretation of the results in the context of other evidence. | 4. Discussion |
|  | 23b | Discuss any limitations of the evidence included in the review. | 4. Discussion |
|  | 23c | Discuss any limitations of the review processes used. | 4. Discussion |
|  | 23d | Discuss implications of the results for practice, policy, and future research. | 4. Discussion |
| **OTHER INFORMATION** | | |  |
| Registration and protocol | 24a | Provide registration information for the review, including register name and registration number, or state that the review was not registered. | 2.1 Data sources and search strategy |
|  | 24b | Indicate where the review protocol can be accessed, or state that a protocol was not prepared. | 2.1 Data sources and search strategy; Supplementary Table S2 |
|  | 24c | Describe and explain any amendments to information provided at registration or in the protocol. | 2.1 Data sources and search strategy; Supplementary Table S2 |
| Support | 25 | Describe sources of financial or non-financial support for the review, and the role of the funders or sponsors in the review. | Funding |
| Competing interests | 26 | Declare any competing interests of review authors. | Conflict of interest |
| Availability of data, code and other materials | 27 | Report which of the following are publicly available and where they can be found: template data collection forms; data extracted from included studies; data used for all analyses; analytic code; any other materials used in the review. | Data availability statement |

**Table S2. Protocol-related details and post-registration clarifications**

| **Item** | **A priori review plan / registration content** | **Final manuscript content** | **Clarification** |
| --- | --- | --- | --- |
| **Review objective** | To evaluate the safety profile of roxadustat in randomized controlled trials of anemia in chronic kidney disease, with emphasis on thrombotic and clinically relevant adverse outcomes. | The final review evaluated thrombotic outcomes and mortality associated with roxadustat in adults with CKD receiving placebo- or ESA-controlled treatment. | The final manuscript retained the same overall review question and scope as planned, with clearer emphasis on thrombotic safety and mortality in response to peer review. |
| **Population and study design** | Adults with CKD; randomized controlled trials comparing roxadustat with placebo or active ESA comparators. | Parallel-group RCTs enrolling adults with CKD, including both dialysis-dependent and non-dialysis-dependent populations; comparators included placebo or active ESAs. | No substantive change was made. The manuscript provides a more explicit PICOS-based description for clarity. |
| **Primary outcome** | Main outcomes included vascular access thrombosis, arteriovenous fistula thrombosis, and arteriovenous graft thrombosis. | VAT was analyzed as the primary outcome throughout the final manuscript. | VAT was listed in the registered PROSPERO record as a main outcome and remained the primary outcome in the final manuscript. |
| **Secondary outcomes** | Additional outcomes included venous thromboembolism, deep vein thrombosis, pulmonary embolism, all-cause mortality, and discontinuation due to adverse events. | Secondary outcomes were all-cause mortality, any venous thromboembolism (VTE), and adverse events leading to treatment discontinuation. | The final manuscript operationalized the registered additional thrombotic outcomes by analyzing any VTE as a composite of DVT and PE, while retaining all-cause mortality and discontinuation due to adverse events as secondary outcomes. |
| **Planned subgroup analyses** | Clinical heterogeneity by dialysis status and comparator type was considered relevant to the review question, although specific subgroup categories were not detailed in the public PROSPERO record. | Subgroup analyses were conducted by comparator type (ESA vs placebo) and dialysis status (DD vs NDD). | These analyses were used to explore potential clinical heterogeneity. The revised manuscript further clarifies that VAT findings in NDD patients should be interpreted cautiously because most NDD patients do not have established vascular access. |
| **Amendments after registration** | No amendments affecting the review question, eligibility criteria, or main outcome were made after registration. | The final manuscript includes clarification of outcome definitions, subgroup interpretation, and protocol transparency. | Post-registration changes were limited to clarification of analytic definitions and reporting transparency and did not alter the core review question, eligibility criteria, or main outcome. |

**Table S3 Detailed search strategies for each database**

| PubMed | | |  |
| --- | --- | --- | --- |
| NO. | Elements | Search strategy |  |
| 1 | Population | ("Renal Insufficiency, Chronic"[Mesh]) OR (Chronic Renal Insufficiencies[Title/Abstract]) OR (Renal Insufficiencies, Chronic[Title/Abstract]) OR (Chronic Kidney Insufficiency[Title/Abstract]) OR (Chronic Kidney Insufficiencies[Title/Abstract]) OR (Kidney Insufficiencies, Chronic[Title/Abstract]) OR (Chronic Renal Insufficiency[Title/Abstract]) OR (Kidney Insufficiency, Chronic[Title/Abstract]) OR (Chronic Kidney Diseases[Title/Abstract]) OR (Chronic Kidney Disease[Title/Abstract]) OR (Disease, Chronic Kidney[Title/Abstract]) OR (Diseases, Chronic Kidney[Title/Abstract]) OR (Kidney Disease, Chronic[Title/Abstract]) OR (Kidney Diseases, Chronic[Title/Abstract]) OR (Chronic Renal Diseases[Title/Abstract]) OR (Chronic Renal Disease[Title/Abstract]) OR (Disease, Chronic Renal[Title/Abstract]) OR (Diseases, Chronic Renal[Title/Abstract]) OR (Renal Disease, Chronic[Title/Abstract]) OR (Renal Diseases, Chronic[Title/Abstract]) | 201802 |
| 2 | Intervention | ("roxadustat" [Supplementary Concept]) OR (FG-4592[Title/Abstract]) OR (FG4592[Title/Abstract]) | 327 |
| 3 | Study design | (randomized controlled trial[pt] OR controlled clinical trial[pt] OR randomized[tiab] OR placebo[tiab] OR "clinical trials as topic"[mesh:noexp] OR randomly[tiab] OR trial[tiab]) NOT (animals[mh] NOT humans[mh]) |  |
| 4 |  | #1 AND #2 AND #3 | 154 |
| Embase | | |  |
| NO. | Elements | Search detail |  |
| 1 | Population | 'chronic kidney failure'/exp OR 'renal insufficiency, chronic':ab,ti OR 'chronic renal insufficiencies':ab,ti OR 'renal insufficiencies, chronic':ab,ti OR 'chronic kidney insufficiency':ab,ti OR 'chronic kidney insufficiencies':ab,ti OR 'kidney insufficiencies, chronic':ab,ti OR 'chronic renal insufficiency':ab,ti OR 'kidney insufficiency, chronic':ab,ti OR 'chronic kidney diseases':ab,ti OR 'chronic kidney disease':ab,ti OR 'disease, chronic kidney':ab,ti OR 'diseases, chronic kidney':ab,ti OR 'kidney disease, chronic':ab,ti OR 'kidney diseases, chronic':ab,ti OR 'chronic renal diseases':ab,ti OR 'chronic renal disease':ab,ti OR 'disease, chronic renal':ab,ti OR 'diseases, chronic renal':ab,ti OR 'renal disease, chronic':ab,ti OR 'renal diseases, chronic':ab,ti | 162,578 |
| 2 | Intervention | 'roxadustat'/exp OR 'roxadustat':ab,ti OR 'fg 4592':ab,ti OR 'fg4592':ab,ti | 899 |
| 3 | Study design | 'randomized controlled trial'/exp OR 'clinical trial'/exp OR 'randomization'/exp OR 'placebo'/exp OR 'random*':ab,ti OR 'blind*':ab,ti OR 'placebo':ab,ti |  |
| 4 |  | #1 AND #2 AND #3 | 354 |
| Cochrane | | |  |
| NO. | Elements | Search detail |  |
| 1 | Population | MeSH descriptor: [Renal Insufficiency, Chronic] explode all trees | 10056 |
| 2 | Intervention | ("Kidney Diseases, Chronic" OR "Chronic Kidney Disease" OR "Renal Disease, Chronic" OR "Chronic Renal Disease" OR "Chronic Renal Diseases" OR "Diseases, Chronic Kidney" OR "Chronic Kidney Diseases" OR "Kidney Disease, Chronic" OR "Renal Diseases, Chronic" OR "Diseases, Chronic Renal" OR "Disease, Chronic Kidney" OR "Disease, Chronic Renal" OR "Chronic Kidney Insufficiencies" OR "Kidney Insufficiencies, Chronic" OR "Chronic Renal Insufficiency" OR "Kidney Insufficiency, Chronic" OR "Chronic Kidney Insufficiency" OR "Chronic Renal Insufficiencies" OR "Renal Insufficiencies, Chronic"):ti,ab,kw | 22724 |
| 3 | Intervention Total | #1 OR #2 | 25025 |
| 4 | Intervention | (Roxadustat OR ASP1517 OR FG4592 OR FG-4592):ti,ab,kw | 240 |
| 5 | Study design | MeSH descriptor: [Randomized Controlled Trial] explode all trees OR ("Randomized Controlled Trial" OR "controlled clinical trial" OR "Clinical Trial" OR randomized OR random OR randomly OR randomised OR placebo OR trial OR groups):ti,ab,kw |  |
| 6 |  | #3 AND #4 AND #5 | 152 |
| Web of Science | | | |
| NO. | Elements | Search detail |  |
| 1 | Population | TS=(Renal Insufficiency, Chronic) OR AB=(Chronic Renal Insufficiencies OR Renal Insufficiencies, Chronic OR Chronic Kidney Insufficiency OR Chronic Kidney Insufficiencies OR Kidney Insufficiencies, Chronic OR Chronic Renal Insufficiency OR Kidney Insufficiency, Chronic OR Chronic Kidney Diseases OR Chronic Kidney Disease OR Disease, Chronic Kidney OR Diseases, Chronic Kidney OR Kidney Disease, Chronic OR Kidney Diseases, Chronic OR Chronic Renal Diseases OR Chronic Renal Disease OR Disease, Chronic Renal OR Diseases, Chronic Renal OR Renal Disease, Chronic OR Renal Diseases, Chronic) | 175952 |
| 2 | Intervention | TS=(roxadustat) OR AB=(FG-4592 OR FG4592) | 1067 |
| 3 | Study design | TS=(Randomized Controlled Trial OR Clinical Trial OR Placebo OR Random* OR Blind* OR Trial) |  |
| 4 |  | #1 AND #2 AND #3 | 400 |

**Figure S1 Leave-one-out sensitivity analysis
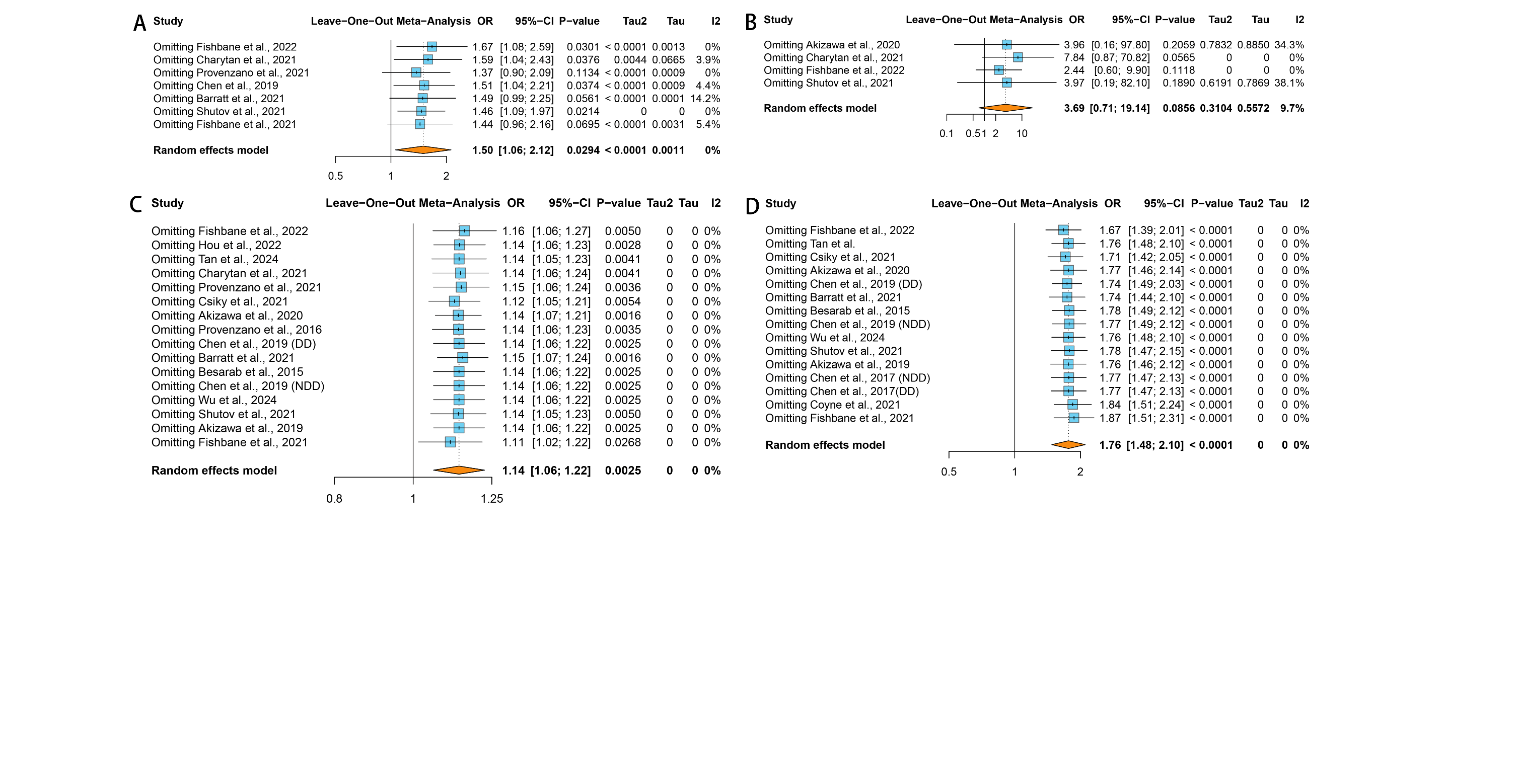
**

Note: (A) VAT; (B) any VTE; (C) all-cause mortality; (D) AEs leading to treatment discontinuation; OR, odds ratio; 95% CI, 95% confidence interval.

**Figure S2 Forest plots of fixed effect**
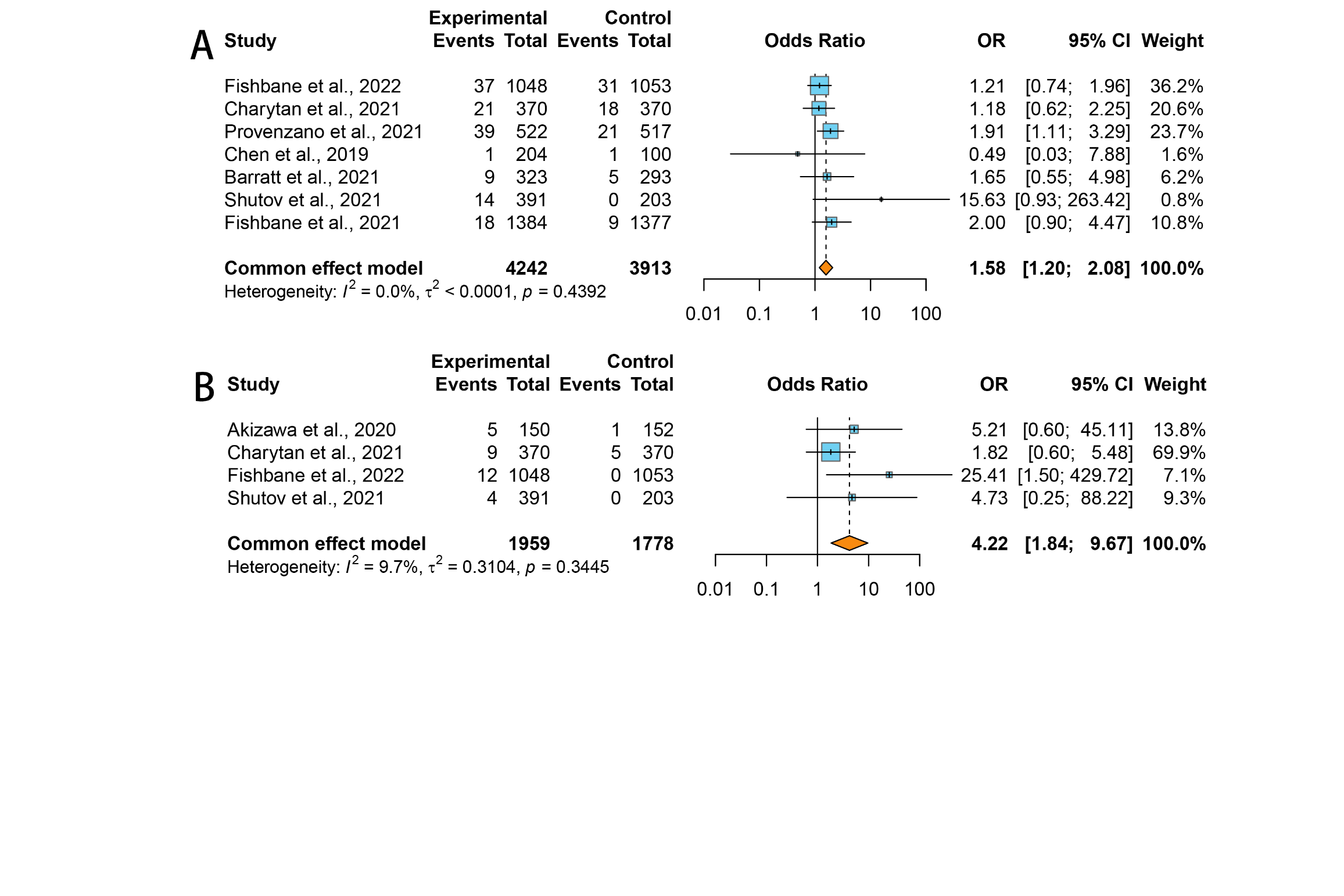


Note: (A) VAT; (B) any VTE; OR, odds ratio; 95% CI, 95% confidence interval.

**Figure S3 Forest plots of fixed effect**


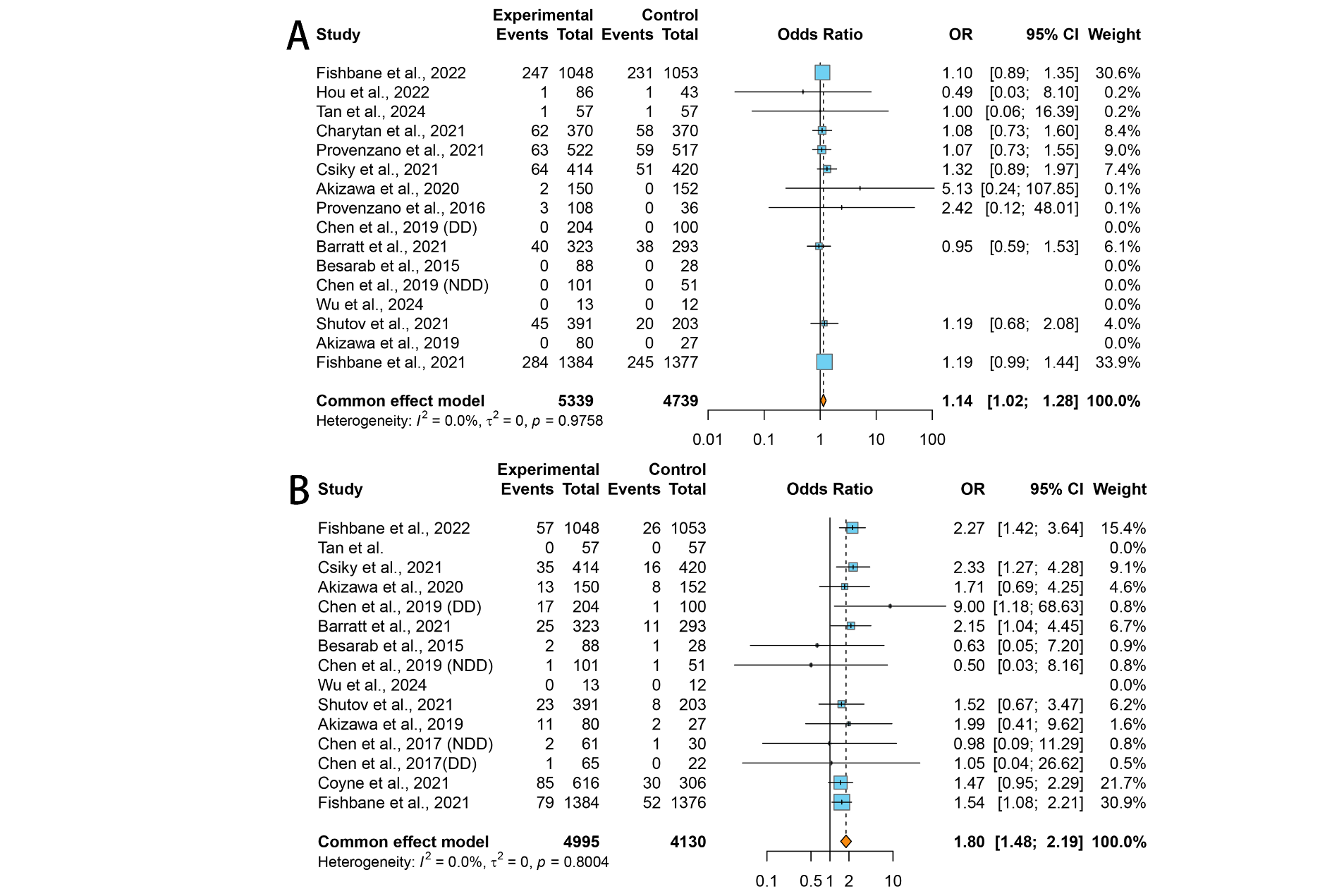


Note: (A) all-cause mortality; (B) AEs leading to treatment discontinuation; OR, odds ratio; 95% CI, 95% confidence interval.

**Figure S4 Funnel plot assessing publication bias for all-cause mortality**





**Figure S5 Funnel plot assessing publication bias for AEs leading to treatment discontinuation**
